# Custom foot orthoses improve performance, but do not modify the biomechanical manifestation of fatigue, during repeated treadmill sprints

**DOI:** 10.1007/s00421-020-04427-0

**Published:** 2020-06-30

**Authors:** Olivier Girard, Jean-Benoit Morin, Joong Hyun Ryu, Ken Van Alsenoy

**Affiliations:** 1 School of Human Sciences (Exercise and Sport Science), University of Western Australia, Crawley, Perth, WA Australia; 2 Aspetar Orthopaedic and Sports Medicine Hospital, Doha, Qatar; 3 Université Côte D’Azur, LAMHESS, Nice, France; 4 Aspire Academy, Doha, Qatar; 5 Centre for Health, Activity and Rehabilitation Research (CHEAR), Queen Margaret University, Edinburgh, Musselburgh, Scotland, UK

**Keywords:** Insoles, Instrumented treadmill, Running kinematics, Leg-spring behaviour

## Abstract

**Purpose:**

We determined the effect of custom foot orthotics manufactured from ethyl-vinyl acetate (EVA) and expanded thermoplastic polyurethane (TPU) materials, both compared to a control condition (CON; shoes only) during repeated sprints on running mechanical alterations.

**Methods:**

Eighteen males performed eight, 5-s sprints with 25-s recovery on an instrumented sprint treadmill in three footwear conditions (EVA, TPU and CON). Mechanical data consisted of continuous (step-by-step) measurement of running kinetics and kinematics, which were averaged for each sprint for further analysis.

**Results:**

Distance ran in 5 s decreased from first to last sprint (*P* < 0.001), yet with higher sprints 1–8 values for both EVA (*P* = 0.004) and TPU (*P* = 0.018) versus CON. Regardless of footwear condition, mean horizontal forces, step frequency, vertical and leg stiffness decreased from sprint 1 to sprint 8 (all *P* < 0.001). Duration of the propulsive phase was globally shorter for both EVA (*P* = 0.002) and TPU (*P* = 0.021) versus CON, while braking phase duration was similar (*P* = 0.919). In the horizontal direction, peak propulsive (*P* < 0.001), but not braking (*P* = 0.172), forces also decreased from sprint 1 to sprint 8, independently of conditions.

**Conclusion:**

Compared to shoe only, wearing EVA or TPU custom foot orthotics improved repeated treadmill sprint ability, yet provided similar fatigue-induced changes in mechanical outcomes.

## Introduction

The capacity to reiterate “all out” efforts with incomplete recoveries (i.e. repeated sprint ability) depends, not only, on metabolic but also neuro-mechanical factors (Girard et al. 2011a). Identifying the onset of fatigue through ground reaction forces (GRF) monitoring during repeated sprint exercise (RSE) may be helpful in providing early warnings of increased injury risk in many team sports. An important limitation of existing RSE literature is the lack of continuous measurements of running velocity and GRF including horizontal force production (Girard et al. 2011b, c). In a pioneer instrumented sprint treadmill study, Morin et al. (2011) provided a thorough description of changes in kinetics and kinematics during a multiple-set repeated sprint series. Along with the expected progressive slowing of running velocity, these authors observed both a significant decrease in the capability to produce total (resultant) GRF and a significant and even larger decrease in the ability to apply it with a forward orientation during acceleration.

Runners often use custom foot orthotics (CFOs) in order to rehabilitate from injuries and/or improve comfort. Additional benefits also concern run-induced fatigue reduction through a better preservation of the ability of the body to absorb plantar loading (Lucas-Cuevas et al. 2014a). However, findings have not been unanimous. For instance, wearing foot orthoses altered neuromuscular control during a sub-maximal, 1-h constant-velocity treadmill run and partly protected from the resulting fatigue-induced reductions in rapid force development characteristics of the plantar flexors (Kelly et al. 2011). Contrastingly, CFOs had no positive effect on running mechanical adjustments induced by a 12-min run at ~14.5 km h^−1^ (Patzkowski et al. 2012; Lucas-Cuevas et al. 2014b). Identification of the biomechanical manifestation of fatigue during RSE with versus without CFOs is currently lacking.

Flexible shank-dependent CFOs are derived from a three-dimensional representation of the individual’s foot and are commonly made of ethyl-vinyl acetate (EVA), while the use of more durable thermoplastic polyurethane (TPU) foams is increasingly popular. By increasing tibial accelerations, peak eversion and tibial internal rotation parameters, this new TPU material is thought to promote energy return (Sinclair et al. 2014), in comparison with traditional EVA footwear midsoles, which may translate into more economical movements (i.e. lower oxygen uptake; Sinclair et al. 2016), thereby minimizing fatigue occurrence. In support, running shoes with softer and more resilient midsoles were found to improve running economy by ~1% on average during treadmill running at a speed slightly below the anaerobic threshold (Worobets et al. 2014). Contrasting results have also been observed regarding sub-maximal running at constant pace with softer shoes with reports of either increase (Bosco and Rusko 1983) or unchanged (Nigg et al. 2003) oxygen uptake values. No previous RSE investigation has investigated whether CFOs with different materials, but identical in construction, protect from the biomechanical manifestation of fatigue.

The primary purpose of offering CFOs choices with varying specifications is to promote comfort, reduce injury risk, improve performance or a combination of the three. In the absence of research studying the biomechanical effects of wearing CFOs during RSE, clinicians require evidence of favourable biomechanical or physiological adjustments to support their prescription. A better understanding of the effects of inserts made of different materials will help to determine if repeated sprint ability can be improved and how this is brought about biomechanically. We therefore determined the effect of CFOs manufactured from EVA and TPU materials, both compared to a control condition (CON; shoes only) during repeated sprints on alterations in running kinetics and kinematics, with special reference to horizontal force production. We hypothesised that wearing CFOs will improve repeated sprint ability by mitigating the effects of fatigue on stride pattern, while only subtle running mechanical differences may be observed between inserts.

## Materials and methods

### Participants

Eighteen male well-trained athletes (mean ± SD age, 38.9 ± 5.1 years; stature, 175.3 ± 5.8 cm; body mass, 74.9 ± 7.7 kg; maximal oxygen uptake, 49.1 ± 6.6 ml min^−1^ kg^−1^; maximal aerobic speed, 18.4 ± 1.6 km h^−1^) were recruited for this study. They trained (running and swimming and/or cycling) on average 8.8 ± 3.7 h per week in the 3 months leading up to the data collection with an average weekly running distance of 37.6 ± 26.7 km. Written informed consent was obtained from participants, and the study was approved by Anti-Doping Laboratory Ethics Committee in Qatar (IRB Application Number 2017000201) and conducted according to the Declaration of Helsinki.

### Protocol overview

About 1 week before testing, participants undertook a preliminary session. They were first requested to perform 7–10 short (<5 s) “familiarisation” sprints. After 10 min of recovery, they completed the RSE (see below). On three occasions, participants performed (in a counter-balanced randomised crossover design), at the same time of day (±1 h) and 4–5 days apart, eight 5-s treadmill sprints with 25-s recovery (participants stood on the treadmill) in different footwear conditions: a control session where participants ran with standardised (i.e. only shoe liner inserted) footwear, CFOs made of EVA and TPU. After arrival to the laboratory, CFOs were inserted bilaterally in participants shoes. The participants and the researcher who was directly involved in guiding the session were visually blinded from the CFO materials. The warm-up consisted of 10 min of running at 10 km h^−1^, followed by 15 min of sprint-specific muscular warm-up exercises, and finally 3–5 submaximal sprints before performing the 3 maximal 5-s sprints separated by 2 min of passive rest. In order to prevent any pacing strategy, the best of these 3 trials was used as the 95% criterion score, which was always satisfied. Participants were then allowed 5 min of free cool down prior to testing.

### Footwear

During all running, the participants used neutral-like running shoes (Pearl Izumi N2v2, Colorado, USA) with an average European shoesize of 43.6 ± 1.6, a stack height of 23–24 mm and a heel drop of 4 mm. The two pairs of CFOs used by participants were based on an individual non-weight-bearing 3D scan of the foot using a Delcam iCube scanner (Elinvision, Karmėlava, Lithuania). CFOs were designed by a sport podiatrist with nearly 20 years of experience, using the Orthomodel Pro CAD software (Autodesk, California, USA). Briefly, scans were imported into the software, markers were placed over the heel, first- and fifth metatarsal and medial arch. A base model surface was adjusted to match the contour of the foot using cross-sectional views from the heel to the forefoot. The thickness of the orthotic was arbitrary set to 8 mm in an attempt to maximise the potential of the expanded thermoplastic polyurethane (TPU) beats inside the Infinergy^®^ material (BASF, Ludwigshafen, Germany). All CFOs were direct-milled out of EVA and TPU materials and manually finished to fit inside the shoes. Wear in time between the first and second intervention session was 4.5 ± 2.5 days and 4.6 ± 2.8 days between the second and last intervention sessions. The weight of the three footwear conditions was on average 600.3 ± 32.0 g, 647.3 ± 36.0 g and 681.1 ± 35.7 g for the shoes with its original liners (CON), with the custom EVA orthoses (EVA) and with the custom TPU orthoses (TPU), respectively.

### Instrumented sprint treadmill

The sprints were performed on an instrumented motorised treadmill (ADAL3D-WR, Medical Development—HEF Tecmachine, France). Briefly, it was mounted on a highly rigid metal frame, set at 0 ° incline, fixed to the ground through four piezoelectric force transducers (KI 9077b; Kistler, Winterthur, Switzerland) and installed on a specially engineered concrete slab to ensure maximal rigidity of the supporting ground. This motorised treadmill allows participants to sprint due to the use of a constant motor torque (Morin et al. 2010). This corresponded to the motor torque necessary to overcome the friction on the belt due to participant’s body weight, which was set to 160% of the default torque after preliminary testing. This default torque value was selected for allowing participants to sprint in a comfortable manner and produce their maximal effort without risking loss of balance. It was measured by requiring the subject to stand unmoving at the centre of the treadmill’s belt and by increasing the driving torque until observing a movement of the belt >2 cm over 5 s.

A single-pass waist and a stiff rope (1 cm in diameter, ~2 m length) were used to tether participants to the 0.4-m vertical rail anchored to the wall behind them. When correctly attached, they were required to lean forward in a typical and standardised crouched sprint-start position with their left foot forward. This starting position was used and standardised all along the sprint series. After a 5-s countdown (“5 s, 3–2–1-Go” given by both visual and audio instructions by the same investigator), the treadmill was released, and the belt began to accelerate as subjects applied a positive horizontal force. Repeated sprint ability was assessed from the averaged distance covered over the eight sprints.

### Mechanical variables

Data were continuously sampled at 1000 Hz, and after appropriate filtering (Butterworth 30 Hz low-pass filter), instantaneous data of vertical, net horizontal and total (resultant) GRF were averaged for each support phase (vertical force above 30 N). These data were completed by measurements of the main step kinematic variables: contact time (s), aerial time (s), step frequency (Hz) and step length (m). Finally, peak braking and peak propulsive forces (BW), duration of braking and propulsive phases (s) along with braking and propulsive impulses (N.s) were determined.

A linear spring-mass model of running was used to investigate the main mechanical integrative parameters characterizing the lower limbs behaviour during running. Vertical stiffness (kN m^−1^) was calculated as the ratio of peak vertical forces (N) to the maximal vertical downward displacement of centre of mass (m), which was determined by double integration of vertical acceleration of centre of mass over time during ground contact. Leg stiffness (kN m^−1^) was calculated as the ratio of peak vertical forces to the maximum leg spring compression [maximal vertical downward displacement + *L*_0_ − √$L_{0}^{2}$ – (0.5 × running velocity × contact time)^2^, m], both occurring at mid-stance. Initial leg length (*L*_0_, a proxy of the great trochanter to ground distance in a standing position) was determined from participant’s stature as *L*_0_ = 0.53 × stature (Morin et al. 2005).

### Responses to exercise

Oxygen uptake was recorded during the entire repeated-sprint protocol (from the beginning of the warm-up to the end of the repeated-sprint protocol) following calibration according to manufacturer’s recommendations. Expired ventilation samples were collected by a metabolic cart (JeagerTM Oxycon Mobile, Carefusion, Germany) for determination of oxygen consumption. Heart rate was also monitored (RS800sd; Polar Electro Oy, Finland). The values of oxygen consumption and heart rate were averaged to obtain a single value for each sprint-recovery cycle (i.e. 30 s). The metabolic cart was suspended from the ceiling next to participants, so they did not have to support the additional weight of the system when running. Ratings of perceived exertion (RPE) were recorded using the Borg 6–20 scale (i.e. 6 = no exertion at all, 20 = maximal exertion) exactly 10 s following each sprint.

### Statistical analysis

Values are presented as mean ± SD. Two-way repeated-measures analysis of variance (ANOVAs) [time (sprints 1–8) × condition (CON, EVA, and TPU)] were used to compare investigated variables. To assess assumptions of variance, Mauchly’s test of sphericity was performed using all ANOVA results. A Greenhouse–Geisser correction was performed to adjust the degree of freedom if an assumption was violated, while a Bonferroni post hoc multiple comparison was performed if a significant main effect was observed. For each ANOVA, partial eta-squared (*η*^2^) was calculated as measures of effect size. Values of 0.01, 0.06 and above 0.14 were considered as small, medium and large, respectively. All statistical calculations were performed using SPSS statistical software V.21.0 (IBM Corp., Armonk, NY, USA). The significance level was set at *P* < 0.05.

## Results

**Fig. 1:**
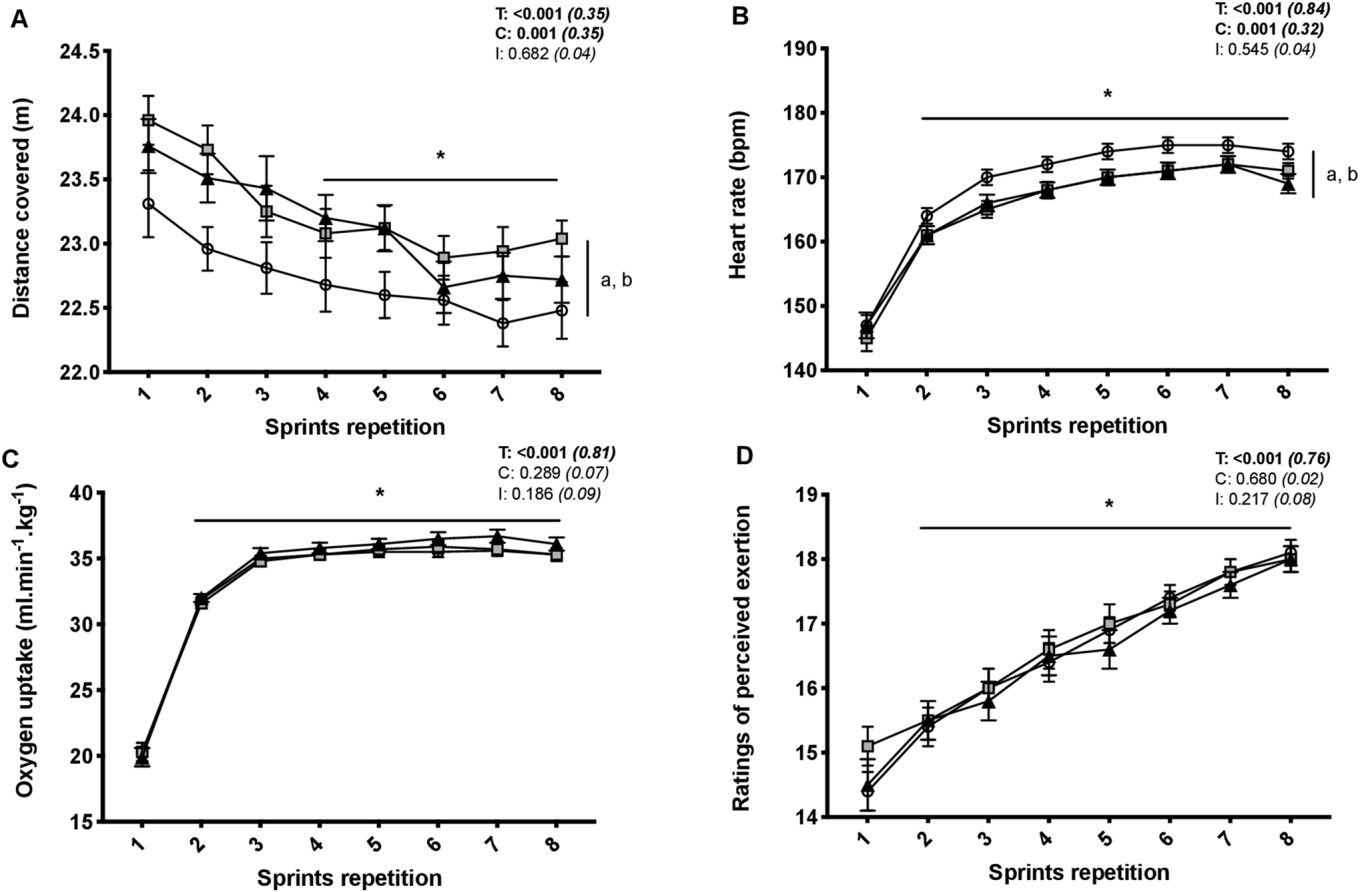
Distance covered (**a**), heart rate (**b**), oxygen uptake (**c**) and ratings of perceived exertion (**d**) during the repeated sprint exercise in three different footwear conditions (CON = shoes only, white circles; EVA = shoes + EVA orthotic, grey squares; TPU = shoes + TPU orthotic, black triangles). Values are mean ± SD (*n* = 18). *C*, *T*, and *I*, respectively, refer to ANOVA main effects of condition, time and interaction between these two factors with *P *value and partial eta-squared into brackets. (Asterisk) significantly different from sprint 1 (all conditions pooled) (*P* < 0.05). **a** CON different from EVA (all sprints pooled) (*P* < 0.05). **b** CON different from TPU (all sprints pooled) (*P* < 0.05)

Distance ran in 5 s decreased from the first to the last sprint (−3.9 ± 3.1%; *P* < 0.001), independent of condition (*P* = 0.682). Averaged distance covered values for the eight sprints were higher for both EVA (23.3 ± 1.4 m; *P* = 0.004) and TPU (23.1 ± 1.5 m; *P* = 0.018) than CON (22.7 ± 1.6 m) (Fig. 1). The eight sprints for both EVA and TPU produced lower heart rates (~4 bpm; *P* < 0.001) compared to CON, while oxygen uptake (*P* = 0.280) and ratings of perceived exertion (*P* = 0.680) values did not differ between conditions (Fig. 1).

**Fig. 2:**
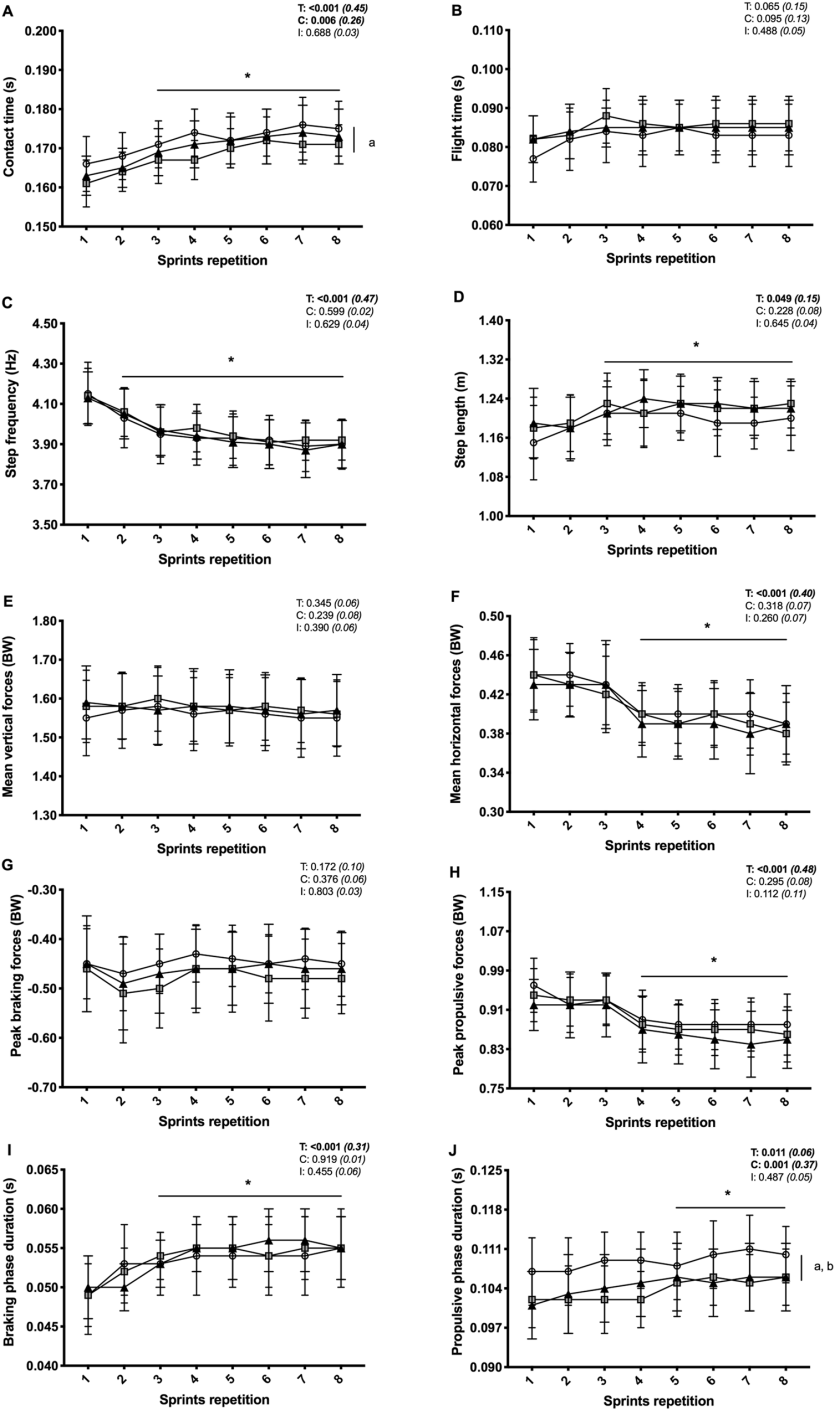
Contact time (**a**), flight time (**b**), step frequency (**c**), step length (**d**), mean vertical force (**e**), mean horizontal force (**f**), peak braking force (**g**), peak propulsive force (**h**), braking phase duration (**i**) and propulsive phase duration (**j**) during the repeated sprint exercise in three different footwear conditions (CON = shoes only, white circles; EVA = shoes + EVA orthotic, grey squares; TPU = shoes + TPU orthotic, black triangles). Values are mean ± SD (*n* = 18). *C*, *T*, and *I*, respectively, refer to ANOVA main effects of condition, time and interaction between these two factors with *P *value and partial eta-squared into brackets. Asterick significantly different from sprint 1 (all conditions pooled) (*P* < 0.05). **a** CON different from EVA (all sprints pooled) (*P* < 0.05). **b** CON different from TPU (all sprints pooled) (*P* < 0.05)

Regardless of the footwear condition, mean horizontal forces (−11.1 ± 5.8%) and step frequency (−5.7 ± 4.8%) decreased from sprint 1 to sprint 8 (*P* < 0.001), while peak and mean vertical forces remained unchanged (*P* > 0.261) (Fig. 2). Contact time lengthened from the first to the last sprint (+9.6 ± 8.7%; *P* < 0.001) as a result of significantly longer braking (+11.5 ± 10.3%; *P* < 0.001) and propulsive (+4.2 ± 4.0%; *P* = 0.031) phases. Averaged contact time values for the eight sprints were also significantly shorter for EVA (−2.3 ± 2.8%; *P* = 0.010), but not TPU (−1.1 ± 3.3%; *P* = 0.432), compared to CON. Specifically, the duration of the propulsive phase was globally shorter for both EVA (−4.7 ± 4.6%; *P* = 0.002) and TPU (−4.0 ± 5.7%; *P* = 0.021) versus CON, while braking phase duration was similar (*P* = 0.919). In the horizontal direction, peak propulsive forces (−8.0 ± 6.7%; *P* < 0.001) decreased from sprint 1 to sprint 8, independently of conditions, but the same was not observed for the braking forces (+2.0 ± 6.7%; *P* = 0.172). Braking impulse (pooled values: −7.7 ± 2.5 vs. −8.8 ± 2.8 N.s; + 22.9 ± 23.8%; *P* = 0.018; *η*^2^ = 0.21) increased and propulsive impulse (pooled values: 51.0 ± 18.0 vs. 48.2 ± 18.3 N.s; −5.4 ± 2.1%; *P* = 0.027;* η*^2^ = 0.24) decreased from the first to the last sprint. The braking impulse was not different between conditions (pooled values: −8.4 ± 2.8 N.s; *P* = 0.377; *η*^2^ = 0.06). The propulsive impulse was globally smaller for both EVA (49.0 ± 7.9 N.s; −3.0 ± 8.0%; *P* = 0.035) and TPU (48.8 ± 8.3 N.s; −3.5 ± 7.9%; *P* = 0.025) than CON (50.5 ± 8.0 N.s).

**Fig. 3:**
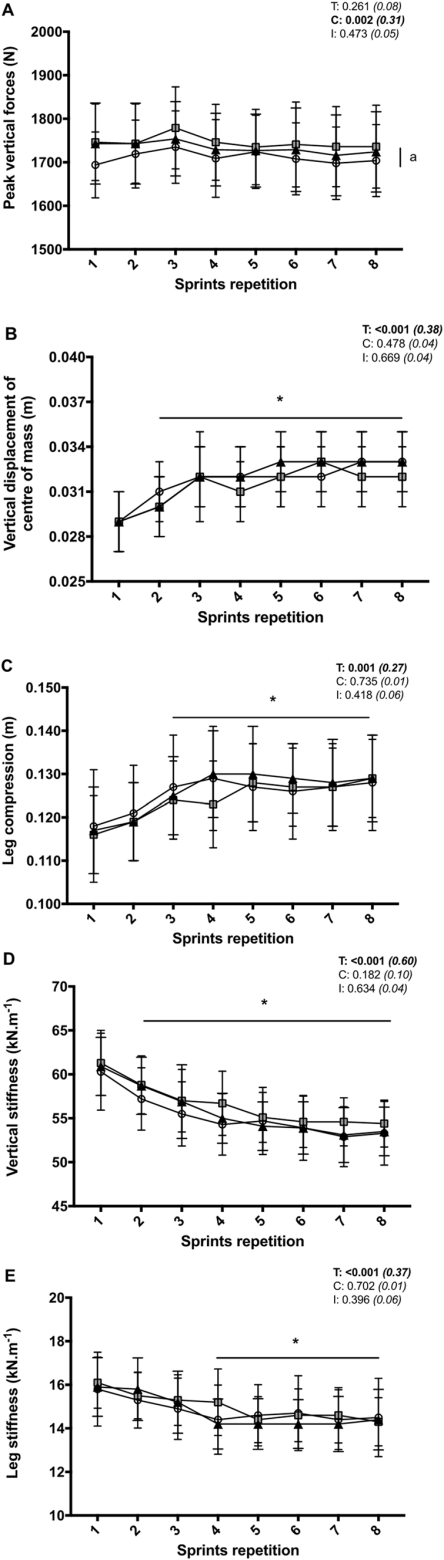
Peak vertical forces (**a**), vertical displacement of the centre of mass (**b**), leg compression (**c**), vertical stiffness (**d**) and leg stiffness (**e**) during the repeated sprint exercise in three different footwear conditions (CON = shoes only, white circles; EVA = shoes + EVA orthotic, grey squares; TPU = shoes + TPU orthotic, black triangles). Values are mean ± SD (*n* = 18). *C*, *T*, and *I*, respectively, refer to ANOVA main effects of condition, time and interaction between these two factors with *P *value and partial eta-squared into brackets. (Asterisk) significantly different from sprint 1 (all conditions pooled) (*P* < 0.05). **a** CON different from EVA (all sprints pooled) (*P* < 0.05)

Irrespective of conditions, vertical (pooled values: −11.7 ± 9.8%) and leg stiffness (−9.5 ± 8.8%) decreased from sprint 1 to sprint 8 (both *P* < 0.001) (Fig. 3). Peak vertical force averaged values for the eight sprints were also significantly larger for EVA (+1.9 ± 2.3%; *P* = 0.008), but not TPU (+1.2 ± 2.4%; *P* = 0.109), compared to CON.

## Discussion

Our main finding was that CFOs improve performance during repeated treadmill sprints, yet with similar positive effects (e.g. ~+ 4% on averaged sprints 1–8 distance covered) for EVA and TPU materials. For the first time with RSE, we investigated the effects of CFOs on the biomechanical manifestation of fatigue. Importantly, no significant interaction was found between sprint number and footwear conditions for any stride mechanical parameter. Despite different resilience characteristics but similar configuration of tested CFOs, the overall results of the present study show that participants who wore CFOs, either made of EVA or TPU materials, produced essentially similar fatigue-induced adjustments in running mechanics during RSE.

Substantial increases in contact time occurred as fatigue developed, leading to a monotonic large decrease in step frequency, while there were non-significant increases in both flight time and step length. Such apparent decreased ability of the lower extremities to tolerate the ground impact observed across sprint repetitions is consistent with previous RSE observations (Girard et al. 2011b, c; Brocherie et al. 2016). Another unique observation was that, compared to CON, duration of propulsive phase was ~ 4–5% shorter for EVA and TPU trials, but was similar for braking phase in all conditions. While peak propulsive forces were similar, for the first time, we demonstrated that wearing insoles during RSE primarily influence the propulsive versus the braking component of the horizontal GRF by shortening the duration of propulsive force application.

There was no significant interaction between time and condition for any of the kinetic and kinematic variables studied. Consistent with previous RSE studies (Morin et al. 2011; Girard et al. 2015; Brocherie et al. 2016), our data showed that reduction in averaged horizontal force production was substantial, while no significant change occurred in the vertical direction. Remarkably, alterations in mean horizontal force production displayed a biphasic pattern with more marked decreases after the third sprint repetition. Following the first few repetitions of a sprint series, the time course of neuromuscular fatigue causing the reduction in performance is due mainly to peripheral fatigue, while central fatigue generally appears later (Collins et al. 2018). Altogether, this reinforces that producing large amounts of horizontal forces to the ground is paramount to better preserve sprint capacity as fatigue develops during RSE, yet wearing CFOs had no effect on this fatigue pattern.

Another novel aspect of this study was to specifically and continuously monitor braking versus propulsive peak forces and phase durations in the horizontal direction, as previously done for single running sprint (Morin et al. 2015; Nagahara et al. 2018). Globally, lengthening of braking versus propulsive phase duration was ~2.5 times larger, and thereby was mainly responsible for progressively longer contact times with sprints repetition. Additionally, peak propulsive but not braking forces decreased with sprint repetitions, with also higher braking and lower propulsive impulses, and followed a similar time course of changes than averaged horizontal force production. This implies that, with fatigue development, runners seem to slow down by pushing less forcefully forward as opposed to braking more in the early stance phase. Interestingly, lengthening of braking and propulsive phase durations (+11.4% and +4.2% from sprint 1 to 8, respectively) was of similar magnitude in elite female Rugby Sevens players undertaking the same RSE (Girard et al. 2020). However, this resulted in significantly lower values for propulsive (−11.9%), but not braking (+1.7%), impulses since alteration in peak braking and propulsive forces was of similar magnitude (~12–13%). This reinforces that biomechanical manifestation of fatigue during RSE are specific to the tested population.

In the present study, similar fatigue-induced changes on spring mass model characteristics occurred while sprinting repeatedly in our three conditions. In line with previous studies where participants did not wear CFOs (Girard et al. 2011b, c; Brocherie et al. 2016), reductions in vertical stiffness and leg stiffness to a lower extent resulted from greater vertical displacement of the centre of mass and leg compression, respectively, since peak vertical forces did not change. Even confronted with larger applied peak vertical forces (albeit only significant for EVA), neither vertical displacement of the centre of mass nor leg compression magnitudes changed when participants wore CFOs, so that vertical and leg stiffness values remained unchanged. Previously, an increase in energy return and/or longitudinal bending stiffness shoe features induced only subtle differences in stride mechanics during overground running at an intensity lower than the ventilatory anaerobic threshold (Flores et al. 2019). Contrastingly, we demonstrated for the first time that EVA, and TPU to a lower extent (non-significant trend), can shorten the contact time and increase peak vertical forces, thereby producing more forceful ground contacts during RSE.

In line with previous research on the effect of orthoses on aerobic cost during a 1-h run (Kelly et al. 2011), we found lower heart rates, but not oxygen uptake values, while wearing CFOs. Interestingly, RPE profile did not differ between trials, probably due the combined effects of improved performance with lower heart rates in EVA and TPU trials. While participants were blinded to shoe wear conditions, a “belief” or expectation effect cannot be overlooked to partly explain these results. This may have in turn, consciously or unconsciously, contributed to maintain motivation to perform maximally during repetition of “all out” efforts. In the absence of measures of task effort awareness (i.e. sense of effort; Christian et al. 2014) and shoe comfort, it is, however, difficult to accept or reject this hypothesis.

Improved performance while wearing CFOs occurred despite the increased footwear weight in EVA and TPU trials (~+ 8 and + 14% compared to CON, respectively). While heavier footwear can be detrimental to mechanical and/or energy cost of distance running (Frederick et al. 1986; Franz et al. 2012), at least under the present circumstances, this confounding factor did not negatively influence repeated sprint ability. Although the sprint-instrumented treadmill is a useful tool for assessment of running biomechanics, caution is needed since measurement of stride mechanical parameters during overground running may differ slightly (Riley et al. 2008). An unexplained finding of our study was that propulsive impulses were ~ 3% lower (braking impulses were comparable), despite better performance, when wearing orthotics. This implies that the present results would need to be confirmed under “real world settings”. Longer sprints, shorter rest periods or a combination of both as well as with environmental perturbations, all exacerbating performance decrements during RSE (Girard et al. 2011a; Brocherie et al. 2016), may derive a greater ergogenic benefit from the addition of CFOs. While no assessment was proposed, it cannot be ruled out that EVA and TPU trials may have been perceived as more comfortable footwear conditions, which would explain better performance maintenance across “all out” efforts. Finally, it remains to be verified if specific changes in GRFs patterns would occur, due to differences in running velocity, during early and late acceleration phase of repeated treadmill sprints (Girard et al. 2015).

## Conclusion

Compared to shoe only, wearing CFOs improve repeated sprint ability, yet with similar positive effects for EVA and TPU materials. There was no noticeable difference in the time course and magnitude of mechanical adjustments for the kinematics, kinetics and spring-mass model parameters during repeated treadmill sprints. While lower extremities behave in a similar manner for the great majority of stride variables, EVA, and TPU to a lower extent, can globally shorten the contact time and increase peak vertical forces compared to CON.

## Abbreviations

ANOVA: Repeated-measures analysis of variance
CFO: Custom foot orthotics
CON: Control condition
EVA: Ethyl-vinyl acetate
GRF: Ground reaction forces
RPE: Ratings of perceived exertion
RSE: Repeated sprint exercise
TPU: Thermoplastic polyurethane
